# Network pharmacology reveals multitarget mechanism of action of drugs to be repurposed for COVID-19

**DOI:** 10.3389/fphar.2022.952192

**Published:** 2022-08-17

**Authors:** Melissa Alegría-Arcos, Tábata Barbosa, Felipe Sepúlveda, German Combariza, Janneth González, Carmen Gil, Ana Martínez, David Ramírez

**Affiliations:** ^1^ Facultad de Ingeniería y Negocios, Universidad de Las Américas, Sede Providencia, Santiago, Chile; ^2^ Departamento de Nutrición y Bioquímica, Facultad de Ciencias, Pontificia Universidad Javeriana, Sede Bogotá, Bogotá, Colombia; ^3^ Department of Molecular Genetics and Microbiology, Biological Sciences Faculty, Pontifical Catholic University of Chile, Santiago, Chile; ^4^ Universidad Externado de Colombia, Departamento de Matemáticas, Bogotá, Colombia; ^5^ Centro de Investigaciones Biológicas Margarita Salas (CIB-CSIC), Madrid, Spain; ^6^ Instituto de Ciencias Biomédicas, Universidad Autónoma de Chile, Santiago, Chile; ^7^ Research Center for the Development of Novel Therapeutic Alternatives for Alcohol Use Disorders, Santiago, Chile

**Keywords:** protein–protein interaction network, protein–drug interaction network, drug repurposing, SARS-CoV-2, COVID-19, network pharmacology, polypharmacology

## Abstract

The coronavirus disease 2019 pandemic accelerated drug/vaccine development processes, integrating scientists all over the globe to create therapeutic alternatives against this virus. In this work, we have collected information regarding proteins from SARS-CoV-2 and humans and how these proteins interact. We have also collected information from public databases on protein–drug interactions. We represent this data as networks that allow us to gain insights into protein–protein interactions between both organisms. With the collected data, we have obtained statistical metrics of the networks. This data analysis has allowed us to find relevant information on which proteins and drugs are the most relevant from the network pharmacology perspective. This method not only allows us to focus on viral proteins as the main targets for COVID-19 but also reveals that some human proteins could be also important in drug repurposing campaigns. As a result of the analysis of the SARS-CoV-2–human interactome, we have identified some old drugs, such as disulfiram, auranofin, gefitinib, suloctidil, and bromhexine as potential therapies for the treatment of COVID-19 deciphering their potential complex mechanism of action.

## Introduction

The recent pandemic is caused by a coronavirus (CoV), which elicits severe acute respiratory syndrome (SARS), an infectious disease named COVID-19. Severe acute respiratory syndrome coronavirus 2 (SARS-CoV-2) emerged in late 2019 in Wuhan, China, where a pneumonia of unknown cause was detected (Zhou et al., 2020). To date, more than 445 million people have been infected and it has caused the death of approximately 5.9 million people worldwide (World Health Organization, 2020). SARS-CoV-2 is a positive-sense RNA virus belonging to the Orthocoronavirinae family, as well as another disease-causing virus such as the Middle East respiratory syndrome (MERS-CoV) or SARS-CoV (Rothan and Byrareddy, 2020).

One of the most important characteristics of SARS-CoV-2 is its transmissibility dynamics because of its highly efficient transmission mechanisms. This infectious agent is usually spread in the respiratory system via inhalation of droplets and/or direct contact, leading to a manifestation of a respiratory dysfunction causing pneumonia in severe infections (Zhang et al., 2020). Interactions between the virus and the host cells are mediated by the direct contact between the spike protein on the virus surface and the ACE2 (angiotensin-converting enzyme 2) membrane receptor in humans, which is widely distributed in many tissues affecting different organs, such as the lungs, heart (Dhakal et al., 2020), kidneys (Fanelli et al., 2020), liver (Wang et al., 2020), and brain (Li et al., 2020), leading to multiorgan failure in severe cases in susceptible individuals (Hamming et al., 2004).

In January 2020, the first genome sequence of SARS-CoV-2 was published (Ren et al., 2020). In addition, to date, there are more than 1,500 entries related to SARS-CoV-2 in the Protein Data Bank (Berman et al., 2000) and 1.4 million assays related to SARS-CoV-2 from the ChEMBL database (Gaulton et al., 2012, 2017), which shows the relevance of this pandemic and the impact in the scientific community, generating a large amount of the data.

Apart from vaccines developed to prevent SARS-CoV-2 transmission and severe COVID-19, the lack of knowledge about potential therapeutic targets, both human and viral, delays the urgent need for effective antiviral development as effective COVID-19 treatments as well as to treat future waves of infection. At present, different approved therapeutic drugs and late-stage clinical drug candidates are being tested for COVID-19 treatment from a drug repurposing approach (Pandey et al., 2020; Sultana et al., 2020).

The basic principle involved in drug repurposing is the use of a previously characterized drug (with a knowledgeable safety assessment, preclinical testing, and formulation development) in other therapies, thus reducing the time required for a new drug to be developed (Talevi and Bellera, 2020). By contrast, it is well known that one of the main goals of systems pharmacology is to integrate and analyze multiomics and clinical data to contribute to the progress of the drug design pipeline, including the identification and characterization of key molecular targets for a given disease such as COVID-19. For instance, the construction of protein–protein, protein–drug, and protein–gene interaction networks, the elucidation of biological and therapeutical effects of the drugs studied, and the determination of possible drug interactions with other targets may lead to a polypharmacological profile according to the interaction with on- and off-targets (Boran and Iyengar, 2010). The ideal scenario would be developing a drug with positive therapeutic effects due to the increased on-target–drug interactions, which would reduce the adverse effects due to the decreased off-target–drug interactions (Iturriaga-Vásquez et al., 2015; Reyes-Parada and Iturriaga-Vasquez, 2016). Thus, the complex and resource-consuming drug design process is shifting from the reductionist 1-target <–> 1-drug model to a new paradigm where the drug polypharmacological profile is widely desired, especially for complex diseases such as COVID-19. The experimental and computational integration of systems biology into polypharmacology broadens the systems pharmacology definition to include the analysis of complex networks at different scales. This provides valuable information to characterize the therapeutic effects of drugs and obtain key elements in the development of new therapeutic alternatives (Zhao and Iyengar, 2012).

The identification of interactions between drugs and target proteins is a key area in genomic drug discovery (Gupta et al., 2021). Since experimental determination of ligand–protein interactions or potential drug–target interactions remains particularly challenging, effective *in silico* prediction methods using drug–target interactions can provide complementary and supporting evidence to experimental studies (Paul et al., 2021). Drug–protein interaction (DPI) networks do not have a bipartite structure when drug–drug and/or protein–protein interactions (PPIs) are present in the network (Galan-Vasquez and Perez-Rueda, 2021). When those interactions are not present in the network, the structure is not bipartite (Janga and Tzakos, 2009; Vogt and Mestres, 2010). In the case of SARS-CoV-2, several databases (such as ClinicalTrials, ChEMBL, DrugBank, and PubChem) contain relevant data to analyze bioactive ligand–protein interactions. In addition, with all the publications related to the COVID-19 pandemic published since late 2019, it is possible to obtain and characterize the SARS-CoV-2 interactome. In this work, we have collected SARS-CoV-2 interactome information from different research articles and databases. We characterize virus–human, virus–virus, and human–human protein interactions relevant to COVID-19. The data were analyzed using network pharmacology including reported drugs (from ChEMBL) that present activity against the identified proteins. With these data, we proposed consensus PPI and DPI networks involved in this disease and identified the FDA-approved drugs, namely, disulfiram, auranofin, gefitinib, suloctidil, and bromhexine, with polypharmacological profiles as good candidates for drug repurposing to treat COVID-19.

## Materials and methods

### Data curation of Human-SARS-CoV-2 interactome

Three datasets were used to build the human–SARS-CoV-2 interactome. The first source of data was a protein interaction map for 26 of the 29 SARS-CoV-2 proteins, and the human proteins physically interact with the viral proteins developed by Gordon et al. (2020). Thus, the data were retrieved from the Krogan’s Lab website (https://ppi.zoiclabs.io/#/- Last accession date 2021-08-31). The second dataset was obtained from the BioID-based interactome of the SARS-CoV-2 proteome (Samavarchi-Tehrani et al., 2020), which takes into account known interactions for SARS-CoV-2 available in BioGRID77 (version 3.5.188) (Stark et al., 2006). The third dataset was obtained from data available for SARS-CoV-2 proteins in the UniProt database (The UniProt Consortium, 2014).

### Protein–protein interactions network (PPI)

We developed a pipeline using Knime analytics platform v4.3.0 (Berthold et al., 2009) to obtain human PPI from the STRING database (Szklarczyk et al., 2021) for each protein obtained in the three datasets previously mentioned. All these data were used to construct a human–virus PPI network using Cytoscape software v3.8.2 (Shannon et al., 2003). Then, both SARS-CoV-2 and human proteins were subjected to enrichment of significant Gene Ontology (GO) terms for biological process, molecular function, and cellular component using the UniProt database crossreference function from the GO database (Ashburner et al., 2000; Carbon et al., 2021) (Supplementary Table S1). Human–human protein interactions were also extracted from the STRING database.

### Drug–protein interactions network (DPI)

DPI network is composed of two types of nodes: different drugs classified as small molecules and molecular targets defined as proteins present in humans or the SARS-CoV-2 virus.

We retrieved this information from the ChEMBL database in the section associated with SARS-CoV-2 (release: ChEMBL29, last accession date 2021-09-12). Then, we selected drugs with the following criteria: “small molecule,” “single-protein,” and report information on “max Phase” (0, 1, 2, 3, and 4), activity as IC_50_ (concentration at half-maximum inhibition) and Ki (inhibitor constant for the protein–inhibitor complex). For drugs that reported more than one IC_50_ or Ki value against a biological target, the highest reported value was considered. In addition, we searched for the target associated with each drug in the “single-protein” section of the ChEMBL database, and only targets from human or SARS-CoV-2 organisms were considered. This was conducted through multiple ChEMBL queries (Nowotka et al., 2017), using an in-house script to automate the process. Then, the data were filtered, and drugs (in phase = 4) that interact with an identified target were kept. Next, we use an activity threshold of 5 µM for IC_50_ and Ki reported activities and kept drugs under this value. We also include drugs with antiviral activity against SARS-CoV-2 recently studied by our group (Ginex et al., 2021). At last, using these data, a DPI network was built using Cytoscape. The pipeline constructed to accomplish the construction of the DPI network is available at https://cutt.ly/HYXDFz6.

### Network analysis

For all the network analysis, R v4.1.1 (R Core Team (2018). R: A language and environment for statistical computing. R Foundation for Statistical Computing, Vienna, Austria. Available online at https://www.R-project.org/) was used to compute topological parameters for each protein and drug in both PPI and DPI networks to understand the relevance of each protein (node) in the network according to the graph theory (Badkas et al., 2021). The topological measures considered were degree, which in a graph is the number of edges that are incident to a vertex. Centrality (also known as eigenvector centrality or prestige score) is a measure of the influence of a node in a network that indicates those nodes connected to other nodes who themselves have high scores (it can be found with the eigenvalues of the adjacency matrix of the graph). Betweenness (also known as betweenness centrality) measures the number of shortest paths that pass through a node, detecting the amount of influence a node has over the flow of information in a graph. Pagerank gives an immediate evaluation of the regulatory relevance of a node; this algorithm was invented by the creators of Google and the idea comes from Markov chains and random walks in the graph. In addition, closeness (also known as closeness centrality) is calculated as the reciprocal of the sum of the length of the shortest paths between a given node and all other nodes in the graph. These topological measures were considered for the network analysis of both interaction networks because they may indicate the regulatory relevance of a node or the capability of communication between nodes in a network, either a protein or a drug. These parameters were obtained and normalized to identify the nodes with the best (top 50%) parameters, with exception of closeness, where the top 1% nodes were selected for further analysis. To identify those nodes that break down the network into two or more components or subnetworks when a specific node is removed, we conducted a network disconnection analysis using graph algorithms (Bang et al., 2019). The code used to perform the previously described network analysis is available at https://cutt.ly/UYXDLgy.

## Results

### PPI network for human and SARS-CoV-2

**FIGURE 1:**
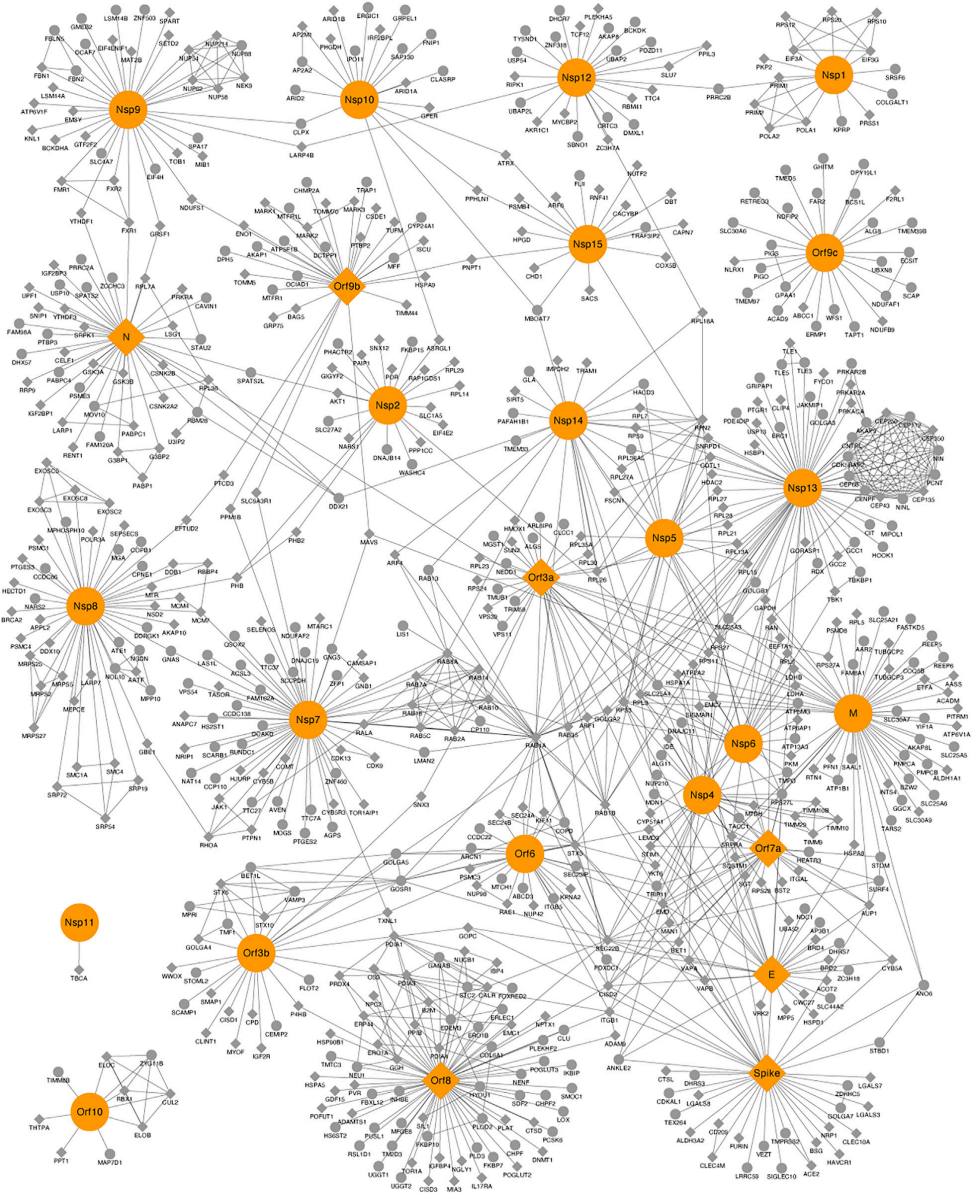
Protein–protein interaction (PPI) network between human–severe acute respiratory syndrome coronavirus 2 proteins. Viral and human proteins are shown in orange and gray, respectively. Proteins with crystallized structures deposited on the Protein Data Bank are shown as diamonds.

The human–SARS-CoV-2 PPI network is composed of 665 proteins, from which 26 proteins correspond to viral proteins, and the remaining 639 proteins are human interacting proteins. The network has 1,176 edges (connections between proteins). Having as a starting point the interactome presented by Krogan’s Lab (Gordon et al., 2020), which identified 332 viral–human interactions for 27 SARS-CoV-2 proteins, we constructed a viral–human protein–protein interactome enriched with an additional 307 human proteins obtained from other two datasets (see the materials and methods section), as well as an enrichment of the human PPIs using STRING database (Figure 1).

The topology parameters (degree, eigenvector centrality, betweenness, pagerank, and closeness) measured for the PPI network suggested a total of 31 proteins, 13 from SARS-CoV-2 (six of these are nonstructural proteins, which share the UniProt ID P0DTC1) and 18 from humans. These proteins met a threshold above 50% for a degree, eigenvector centrality, betweenness, and pagerank, as well as a threshold above 99% for closeness (Supplementary Table S2). Proteins were defined as top proteins because of their importance for the network connection, central role in protein communication, and potential regulators of the underlying biological process (Scardoni et al., 2015). The intersection of these proteins according to each topological parameter is represented in Supplementary Figure S1, where it is observed that there are 12 human proteins (CDK5RAP, CENPF, CEP112, CEP135, CEP250, CEP350, CEP43, CEP68, CNTRL, NIN, NINL, and PCNT) that are in the top 50% according to the eigenvector centrality parameter, and the CEP protein family is an active composition element of the centrosome, playing a key role in centriole biogenesis and cell cycle progression control (Kumar et al., 2013). Five human proteins (CISD2, RPL3, RPS27, RPS3, and SEC22B) and one viral protein (E) are in the top 50% according to degree and betweenness, and four viral proteins (N, Nsp8, Nsp9, and Orf8) are in the top 50% according to pagerank, degree, and betweenness. The M and Nsp7 viral proteins are in the top 50% according to closeness, pagerank, degree, and betweenness, and the human protein RAB1A and the viral protein Nsp14 are in the top 50% according to closeness and betweenness. Orf9b and Orf9c viral proteins are in the top 50% according to pagerank. Nsp13 is in the top 50% according to pagerank, eigenvector centrality, degree, and betweenness. The spike viral protein is in the top 50% according to pagerank and degree, and the viral protein Nsp12 is in the top 50% according to pagerank and betweenness (Supplementary Table S2).

The subnetwork composed of the 31 top proteins is presented in Supplementary Figure S2, where it can be observed that the viral proteins Nsp7, Nsp13, Nsp14, Orf8, M, E, and Spike are the most connected with human top proteins. The viral protein Nsp13 interacts with 12 human proteins (CNTRL, CEP68, CEP43, CEP350, CEP250, CEP135, CEP112, CENPF, CDK5RAP2, PCNT, NINL, and NINL); some of them belong to the CEP family and are localized in the centrosome, Golgi, and cytosol as shown by affinity purification–mass spectrometry results (Miserey-Lenkei et al., 2021). In COVID-19, Nsp13 damages physiological interactions in the structure of cilia through centrosome binding, disrupting the interaction between the centriole and cilia, leading to deciliation (Najafloo et al., 2021). Our analysis allows us to observe that these proteins are quite interconnected and that they are very important for the PPI network. Therefore, modulating one or more of these proteins could exert a significant effect on the interaction of the virus SARS-CoV-2 with the human host cells.

To identify key nodes using other parameters, we conducted a disconnection analysis using graph algorithms. The main idea is to remove a node from the network (Figure 1) and evaluate what happens to it. The disconnection analysis resulted in a total of 24 proteins, 2 human (RAB8A, which interacts with NSP7, and PRRC2B, which interacts with NSP12) and 22 viral proteins, which disconnected the graph in at least two components (Supplementary Table S3). There were six viral proteins common in the results of both the topological and disconnection analysis (Table 1).

**TABLE 1:** Proteins of the protein–protein interaction network that resulted as key proteins from the topological analysis and generated a graph disconnection in the cutoff analysis.

Uniprot ID	Gene name	Organism
P0DTC8	Orf8	SARS-CoV-2
P0DTD2	Orf9b	SARS-CoV-2
P0DTC4	E	SARS-CoV-2
P0DTC5	M	SARS-CoV-2
P0DTC9	N	SARS-CoV-2
P0DTC2	Spike	SARS-CoV-2

### DPI network

**FIGURE 2:**
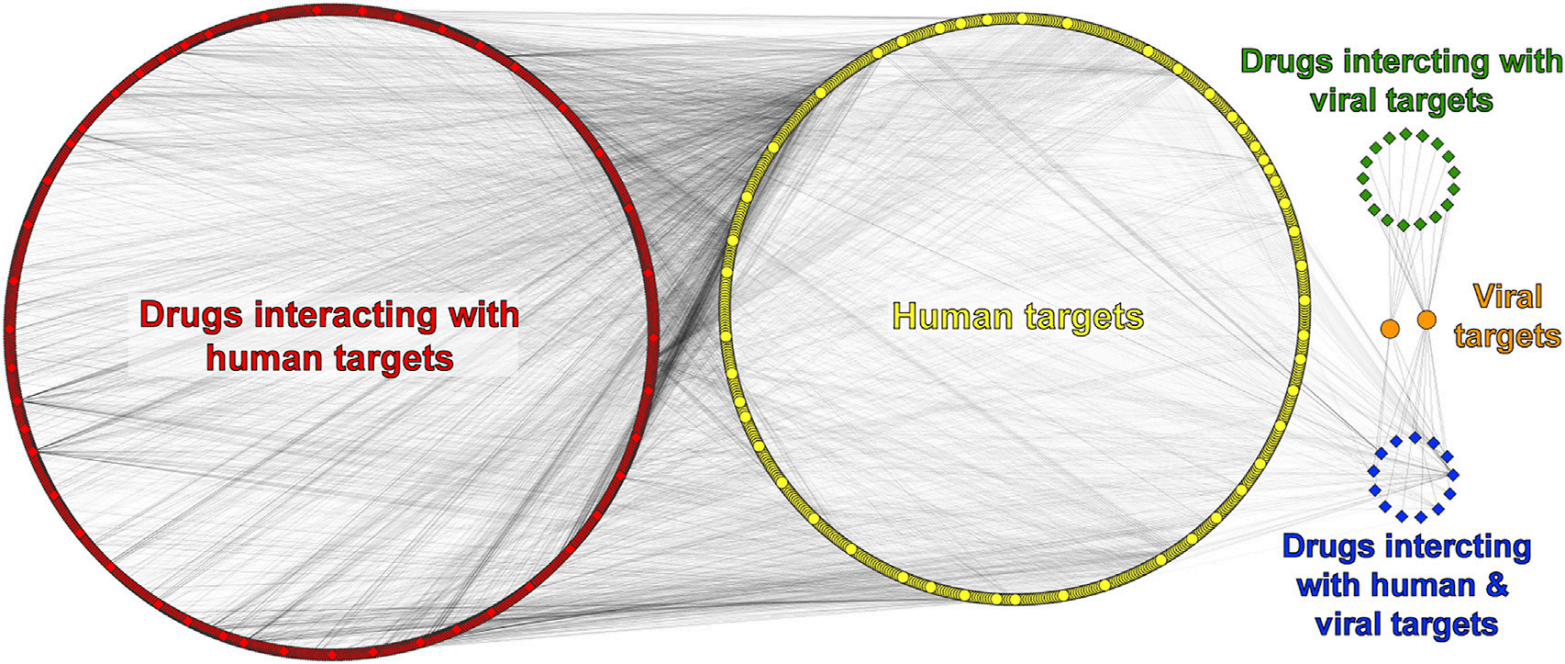
Drug–protein interaction (DPI) network. Human/viral proteins and drugs are shown as circles and diamonds, respectively.

There are 8,208 compounds associated with the SARS-CoV-2 virus in the ChEMBL database. Our first selection considered only drugs/compounds labeled as “small molecule,” and that interacts with a target labeled as “single-protein,” as well as with reported information on both “max Phase” (0, 1, 2, 3, or 4) and experimental activity (IC_50_ and Ki), which reduced our data set to 1999 drugs/compounds. For these 1,999 molecules, there are 1,279 associated targets from human or SARS-CoV-2 organisms. At last, considering only phase = 4 drugs (marketed drugs), activity threshold IC_50_ or Ki ≤ 5,000 nM, and adding reported drugs from literature, the total data set was 868 drugs and 683 targets. To visualize the data, we built a DPI network that had a total of 1,549 nodes (corresponding to proteins and drugs) and 5,017 edges. Moreover, we added five labels to identify each node: 1) yellow for human targets, 2) orange for viral targets, 3) red for drugs interacting with human targets, 4) green for drugs interacting with viral targets, and 5) blue for drugs that interact with both viral and human targets (Figure 2).

In the DPI network (Figure 2), there are 680 human proteins (yellow) and 838 drugs (red) that interact with 499 of those human proteins. The remaining 181 human proteins interact with both groups of drugs at the same time (red and blue). The drug information obtained from the ChEMBL database does not allow us to differentiate which specific viral protein could be interacting with a given drug, unlike the PPI network, which does have information on specific viral proteins. Therefore, only two viral proteins are represented in the DPI network (orange), the replicase polyprotein 1 ab (R1AB-SARS2) and spike protein from SARS-CoV-2. Information from both PPI and DPI networks was merged, and 28 common human proteins were obtained (Supplementary Figure S3). Of these 28 common proteins found, we can highlight some that interact with more than one viral protein. For example, CISD2 reports eight interactions with viral proteins such as Orf3, Orf6, Orf7, Orf8, Nsp4, M, E, and Spike. CYP51A1 has interactions with three viral proteins, namely, Orf3, Nsp6, and E, and both HDAC2 and TBK1 proteins present two interactions with viral proteins. The first one is reported to interact with Nsp5 and Nsp13, and the second one with Nsp13 and Nsp6 as shown in Supplementary Figure S3.

**FIGURE 3:**
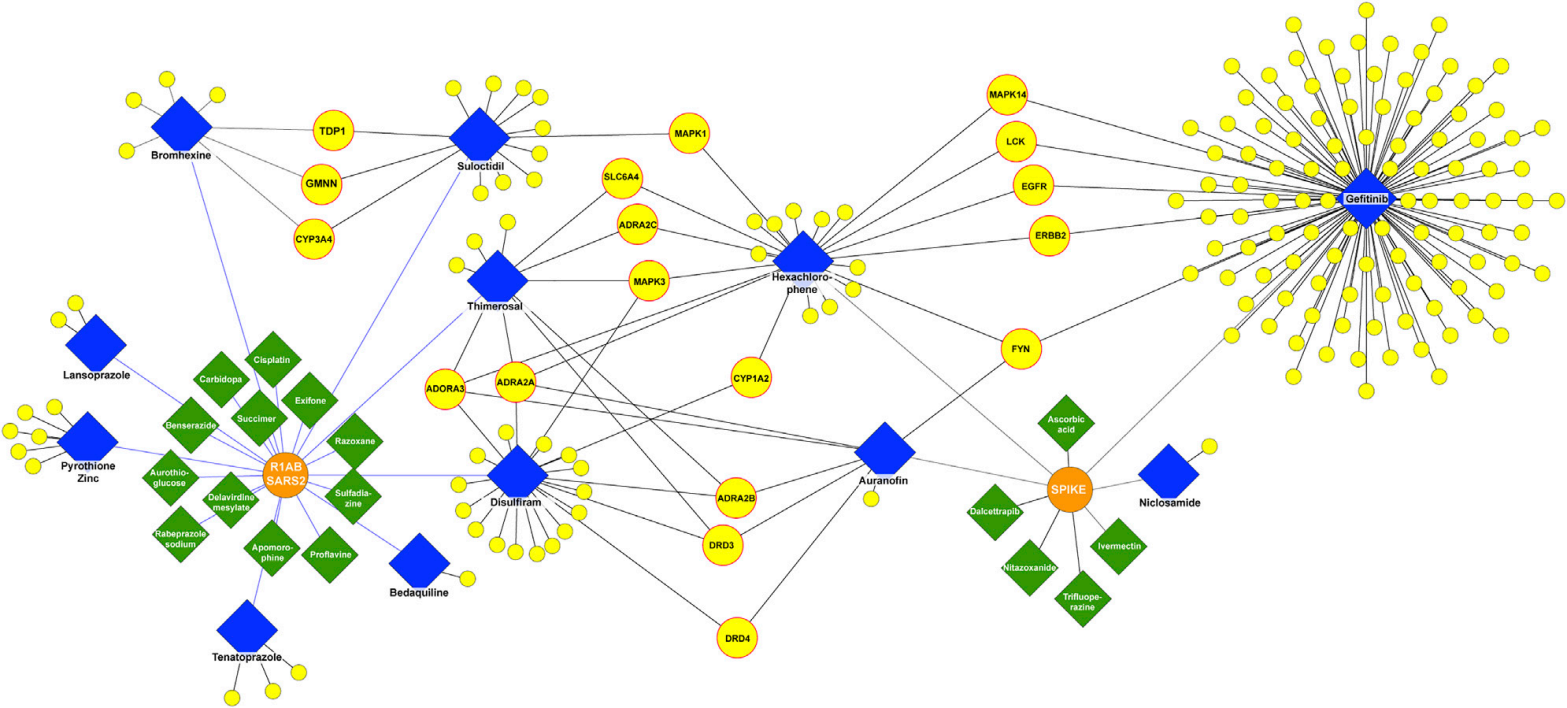
DPI subnetwork. Human and viral proteins are shown as yellow and orange circles, respectively. Drugs that interact only with viral targets are shown as green diamonds and drugs that interact with both viral and human targets as blue diamonds. Yellow circles with a red border represent human proteins interacting with at least two drugs, which also interact with one or more viral proteins.

**FIGURE 4:**
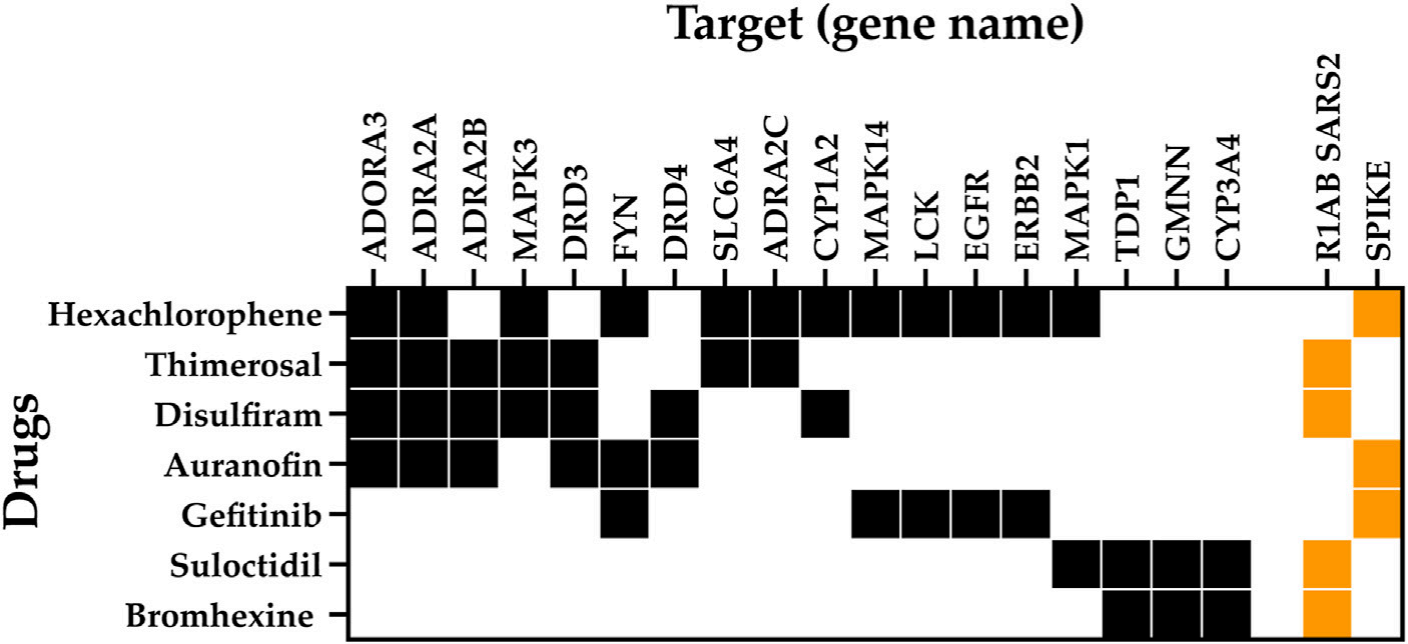
Multitarget interaction profile of the potential antiviral drug. Diagram summarizing drug interactions to key human proteins (black) and viral proteins (orange).

With the filters applied to our pipeline, we found 17 drugs (green) interacting with both R1AB-SARS2 and spike viral proteins (Figure 2), from which 12 interact with R1AB-SARS2 and 5 drugs (ivermectin, ascorbic acid, dalcetrapib, nitazoxanide, and trifluoperazine) are reported to interact with the spike protein (Supplementary Table S4). The most relevant information obtained with the DPI networks was the identification of 13 drugs (blue) that interacts simultaneously with both viral and human proteins (Figure 2), which gives an indication that these drugs may have an antiviral potential because of their polypharmacological profile. In Figure 3, a DPI subnetwork is presented, where reported interactions of viral proteins with different drugs are shown in more detail. We focus on multitarget drugs that interact with both virus and human proteins (blue diamonds); among which, we can find tenatoprazole, lansoprazole, pyrothione zinc, bedaquiline, disulfiram, auranofin, thimerosal, hexachlorophene, gefitinib, suloctidil, and bromhexine. The human proteins (yellow) that interact with at least two drugs are highlighted with red borders. These proteins were considered key human targets because of their ability to be modulated by multitarget drugs that also interact with viral proteins. This viral/human polypharmacological effect could be the key behind its antiviral activity and is summarized in the multitarget interaction profile shown in Figure 4.

These data reveal potentially repurposable drugs and new mechanisms of action for SARS-CoV-2 infection and COVID-19 treatment. Among the key targets, we found the adenosine A3 receptor (ADORA3), which is overexpressed in inflammatory cells. ADORA3 agonists induce downregulation of the NFkB signaling pathway, resulting in decreased TNF-α levels by monocytes and synoviocytes resulting in an anti-inflammatory effect. Adenosine has been proposed with the potential to act in acute lung injury as a local anti-inflammatory (Caracciolo et al., 2021). We identified that hexachlorophene, thimerosal, disulfiram, and auranofin interact with ADORA3. In addition, the alpha-2 adrenergic receptors (ADRA2A, ADRA2B, and ADRA2C) play a role in immune cell activity and inflammatory cytokine production. ADRA2 agonists moderate immune and inflammatory processes and improve oxygenation through physiologic respiratory parameters. ADRA2 agonists are believed to reduce mortality related to hyperinflammation and acute respiratory failure in COVID-19 patients, as well as to have immunomodulatory effects by maintaining endothelial junction integrity and reducing immune cell activity even when an inflammatory stimulus is present (Hamilton et al., 2022). We found that ADRA2A interacts with hexachlorophene, thimerosal, disulfiram, and auranofin; ADRA2B interacts with thimerosal, disulfiram, and auranofin; and ADRA2C interacts with hexachlorophene and thimerosal.

Our analysis showed five core proteins from the epidermal growth factor receptor (EGFR)/mitogen-activated protein kinase (MAPK) pathway interacting with the drugs shown in Figure 4: MAPK3, MAPK1, MAPK14, EGFR, and ERBB2. MAPKs are serine-threonine kinases, also known as extracellular signal-regulated kinases (ERKs). These proteins act in a signaling cascade that regulates proliferation, differentiation, and cell cycle progression in response to a variety of extracellular stimuli (Hutcheson et al., 2003) and are considered therapeutic targets for COVID-19 infection because of their involvement in the platelet activation processes leading to inflammation (Asiedu et al., 2021). EGFR and ERBB2 are transmembrane receptor tyrosine kinases from the erbB family and are involved in the EGFR pathway (Liang et al., 2021). It has been found that EGFR may modulate the wound healing response to SARS-CoV and that the EGFR pathway is a prime target for therapeutic interventions to reduce fibrosis after respiratory virus infection (Venkataraman and Frieman, 2017). SARS-CoV-2 has been demonstrated to have a dependence on the EGFR/MAPK signaling pathway. In an *in vitro* characterization of pulmonary fibrosis due to COVID-19, a dual antifibrotic and antiviral activity of EGFR/ErbB inhibitors has been identified in living host lung cells. This validation demonstrates the utility of targeting this pathway by EGFR/ErbB inhibitors, to achieve a downregulation of ERKs in COVID-19 treatment (Vagapova et al., 2021). In our analysis, MAPK3 interacts with disulfiram, hexachlorophene, and thimerosal; MAPK1 interacts with hexachlorophene and suloctidil; and EGFR, ERBB2, and MAPK14 interact with gefitinib and hexachlorophene.

Other targets found with our network pharmacology pipeline were the proto-oncogene tyrosine–protein kinase Fyn (FYN) and the Cytochrome P450 1A2 (CYP1A2). FYN interacts with gefitinib, hexachlorophene, and auranofin. Transcriptomic analysis suggests that FYN interacts with Spike in the infection process of SARS-CoV-2. In addition, there is evidence proving that the virus causes FYN dysregulation, an event linked with Αβ accumulation, Tau hyperphosphorylation, and innate immunity during COVID-19 (Vavougios et al., 2021). CYP1A2 is involved in drug metabolism and cholesterol synthesis. CYP1A2 showed a decrease of 53% during SARS-CoV-2 infection measured in blood samples from 18 COVID-19 patients, with an inverse correlation with interleukin 6 and C-reactive protein levels (Lenoir et al., 2021). CYP1A2 interacts with disulfiram and hexachlorophene.

Regarding viral proteins, we found that R1AB-SARS2 interacts with thimerosal, disulfiram, suloctidil, bromhexine, and tenatoprazole, and the spike protein interacts with gefitinib, auranofin, and hexachlorophene (Figure 4).

## Discussion

In this work, we described the reconstruction and enrichment of biological and pharmacological knowledge to identify processes impacted by SARS-CoV-2 human infection. From both our PPI and DPI network analyses, which integrate the measure of topological parameters using graph theory mathematical formulations, node disconnection analysis, and network exploration, we found seven approved drugs capable to modulate simultaneously key proteins involved with biological processes associated with SARS-CoV-2 infection and COVID-19 disease (Figure 4).

It is worth mentioning that our PPI network between humans and viruses is the most complete and enriched network among those which have been reported because we integrated data from multiple available interactomes as well as curated databases such as UniProt. To build the DPI network, we retrieved data associated with SARS-CoV-2 available in the ChEMBL database as well as literature and proposed filters that allow obtaining drugs with significant bioactivity using their IC_50_ and Ki values. The results and subsequent analysis of both PPI and DPI networks can be easily reproducible and applicable to any target following the information available at https://github.com/ramirezlab/COVID-protein-drug-network.

The drugs identified in our analysis are not all suitable to be repurposed. For example, hexachlorophene interacts with 12 key human proteins as well as the spike protein but is indicated as a topical antiseptic used as a surgical scrub and skin cleanser and should not be taken orally, which impedes its repurposing for the treatment of COVID-19. Other drugs such as thimerosal (preservative) should be administered via cutaneous or ophthalmic route. Nevertheless, most of the identified drugs can be administrated via an oral route; thus, in addition to their polypharmacological profile, making them good candidates for drug repurposing against COVID-19. Among them, we find disulfiram (used to treat alcohol addiction), auranofin (antirheumatic), gefitinib (used as first-line therapy to treat nonsmall cell lung carcinoma), suloctidil (vasodilator), and bromhexine (mucolytic).

This network pharmacology workflow has enabled us to identify some relevant drugs as candidates for drug repurposing for the treatment of COVID-19 progression based on the viral and human protein interactions observed in our networks. Some of the drugs identified in the present study have in fact been proposed as candidates for the treatment of COVID-19 by different authors, for example, 1) disulfiram, where its uses are associated with a lower risk of COVID-19 (Fillmore et al., 2021); 2) auranofin, which inhibits SARS-CoV-2 replication and attenuates inflammation in human cells (Marzo and Messori, 2020; Rothan et al., 2020); 3) suloctidil, which was recommended for the treatment of COVID-19-induced exacerbation of asthma (Guo et al., 2021); and 4) gefitinib, which was reported to inhibit spike-driven fusion (Braga et al., 2021).

Another example is bromhexine, a mucolytic and antitussive agent, usually low cost and marketed without prescription, which was introduced in the market in 1963 under the brand name Bisolvon^®^ (European Medicines Agency, 2015). It is indicated both for adults and pediatric population, for the management of a wide variety of respiratory conditions in most cases involving alterations in mucus secretion. It is generally well tolerated and has few adverse effects (Chang et al., 2014; Scaglione and Petrini, 2019). We have here found that bromhexine interacts with the R1AB-SARS2 protein, which encodes for 16 nonstructural proteins, and 14 of these are crucial in the process of SARS-CoV-2 replication and promote the activity of the catalytic nonstructural proteins (Malone et al., 2022). It is relevant to highlight that bromhexine has been initially identified as a potent inhibitor (IC_50_ = 0.75 µM) of the transmembrane serine protease 2 (TMPRSS2) of SARS-CoV (Shen et al., 2017), being involved also in the binding and infection (mainly via a nonendocytotic route) of SARS-CoV-2 to host cells (Gil et al., 2020). This interaction was found in our DPI network but not in the subnetwork because it is not significant (with respect to other interactions found where one drug interacts with two or more targets). In fact, this last year much work has been developed to understand the mechanism of action of bromhexine in SARS-CoV-2 infection. Recent studies ruled out that TMPRSS2 inhibition is responsible for the antiviral activity of bromhexine in SARS-CoV-2, as slight antiviral activity is reported in VeroE6 cells, which lack TMPRSS2 in their membranes (Matsuyama et al., 2020). The interactions with other viral proteins here found may be responsible for this direct viral inhibition. In addition, bromhexine reached clinical trials in the first months of the pandemic for hospitalized patients showing a reduction in the mortality rate in patients with COVID-19 (Ansarin et al., 2020).

It is important to note that our research has limitations, mainly given that our research was based on information from public databases with Ki and IC_50_ values reported for other research groups; some of these values were measured in Vero E6 cells, which are not necessarily suitable for all human cell–virus interaction studies. In addition, there may be false positive interactions between drug–protein and protein–protein because we depend on the reported bioactivity values, and in many cases, there is more than one bioactivity data reported for a given molecule, and some values are above or below the threshold defined in this study. Furthermore, the conditions of each reported experiment are not the same; thus, there could exist some skewness in the generated values, and as a result, the interactions reported in this research (DPI and PPI networks) would need further experimental assays for their validation. At last, it is worth noting that before starting new clinical trials, further pharmacokinetic/pharmacodynamic investigations should be considered (e.g., sufficient plasma concentrations and bioavailability) to consider reaching plasma concentrations sufficient to achieve the desired anti-SARS-CoV-2 effect.

## Conclusions

The drugs identified in this work to be repurposed for the treatment of COVID-19 (disulfiram, auranofin, gefitinib, suloctidil, and bromhexine) and other related works reported in recent years show the great need to find efficient therapeutic alternatives to control the development of the infection in the current pandemic, for future waves and even for future infections by other viruses. The known mechanisms of the drugs often fail to explain why they have an antiviral effect against SARS-CoV-2, either by directly interacting with viral proteins or by modulating host cell mechanisms that allow the virus to complete its infection cycle. To find suitable candidates to be repurposed is important to tackle the problem in a different way, shifting the focus of the scientific community from “drug-reductionist” to “drug-holistic” network-based approaches in pharmacology. In this work, we aim to analyze the data that have been collected by several authors over the last few years in public databases and examine all these data within a polypharmacological context. We identified key targets in both SARS-CoV-2 and humans, as well as drugs that we consider worth moving forward to new clinical trials in patients with different characteristics. This will allow us to have a better understanding of how drug–antiviral effect develops and leads to a positive therapeutic effect for the treatment of COVID-19.

## Data Availability

The datasets presented in this study can be found in online repositories. The names of the repository/repositories and accession number(s) can be found below: https://github.com/ramirezlab/COVID-protein-drug-network.
