# Supplementary material for: Network pharmacology reveals multitarget mechanism of action of drugs to be repurposed for COVID-19
Source: Front Pharmacol. 2022 Aug 17;13:952192. doi: 10.3389/fphar.2022.952192 (PMC9424758; doi:10.3389/fphar.2022.952192)
Supplement: Supplementary file 1 [file DataSheet1.pdf]

Supplementary Material

Supplementary Table S1. Functional enrichment and cross-references IDs from multiple databases of proteins included in the networks (Excel file).

Supplementary Table S2. Proteins in the top 50% according with the topological parameters: Degree, Centrality, Betweenness, PageRank, and Closeness

Proteins	Uniprot ID	Organism	Degree	Eigenvector centrality	Betweenness	PageRank	Closeness
RAB1A	P62820	Human			X		X
CISD2	Q8N5K1	Human					X
RPL3	P39023	Human					X
RPS27	P42677	Human					X
RPS3	P23396	Human					X
SEC22B	O75396	Human					X
CDK5RAP2	Q96SN8	Human		X			
CENPF	P49454	Human		X			
CEP112	Q8N8E3	Human		X			
CEP135	Q66GS9	Human		X			
CEP250	Q9BV73	Human		X			
CEP350	Q5VT06	Human		X			
CEP43	O95684	Human		X			
CEP68	Q9NQ79	Human		X			
CNTRL	Q7Z7A1	Human		X			
NIN	Q8N4C6	Human		X			
NINL	Q9Y2I6	Human		X			
PCNT	O95613	Human		X			
M	P0DTC5	SARS-CoV-2	X		X	X	X
Nsp7	P0DTC1	SARS-CoV-2	X		X	X	X
Nsp13	P0DTC1	SARS-CoV-2	X	X	X	X	
N	P0DTC9	SARS-CoV-2	X		X	X	
Nsp8	P0DTC1	SARS-CoV-2	X		X	X	
Nsp9	P0DTC1	SARS-CoV-2	X		X	X	
Orf8	P0DTC8	SARS-CoV-2	X		X	X	
Nsp12	P0DTC1	SARS-CoV-2			X	X	
Spike	P0DTC2	SARS-CoV-2	X			X	
Orf9b	P0DTD2	SARS-CoV-2				X	
Orf9c	P0DTD3	SARS-CoV-2				X	
Nsp14	P0DTC1	SARS-CoV-2			X		X
E	P0DTC4	SARS-CoV-2					X

Supplementary Table S3. Proteins of the PPI network resulting from disconnection analysis.

Uniprot ID	Gene name	Organism	Components generated
P0DTC8	Orf8	SARS-CoV-2	40
P0DTC5	M	SARS-CoV-2	28
P0DTC9	N	SARS-CoV-2	23
P0DTD2	Orf9b	SARS-CoV-2	22
P0DTC2	Spike	SARS-CoV-2	20
P0DTC4	E	SARS-CoV-2	12
P0DTD8	Nsp7	SARS-CoV-2	37
P0DTC1	Nsp8	SARS-CoV-2	25
P0DTC1	Nsp12	SARS-CoV-2	23
P0DTC1	Nsp9	SARS-CoV-2	22
P0DTC1	Nsp2	SARS-CoV-2	18
P0DTC1	Nsp13	SARS-CoV-2	17
P0DTC1	Nsp15	SARS-CoV-2	14
P0DTC1	Nsp10	SARS-CoV-2	14
P0DTC3	Orf3a	SARS-CoV-2	13
P0DTF1	Orf3b	SARS-CoV-2	11
P0DTC6	Orf6	SARS-CoV-2	10
P0DTC1	Nsp1	SARS-CoV-2	8
P0DTC7	Orf7a	SARS-CoV-2	7
P0DTC1	Nsp14	SARS-CoV-2	6
P0DTC1	Nsp4	SARS-CoV-2	6
P0DTC1	Nsp6	SARS-CoV-2	4
P61006	RAB8A	Human	2
Q5JSZ5	PRRC2B	human	2

Supplementary Table S4. Drugs that interact only with viral targets (shown as green diamonds in the DPI network).

ChEMBL ID Drug	Drug name	Viral protein name	Uniprot ID Virus
CHEMBL444186	Razoxane	Replicase polyprotein 1ab	P0DTD1
CHEMBL329522	Exifone	Replicase polyprotein 1ab	P0DTD1
CHEMBL1201073	Succimer	Replicase polyprotein 1ab	P0DTD1
CHEMBL2068237	Cisplatin	Replicase polyprotein 1ab	P0DTD1
CHEMBL1616	Apomorphine	Replicase polyprotein 1ab	P0DTD1
CHEMBL1200930	Rabeprazole	Replicase polyprotein 1ab	P0DTD1
CHEMBL1201236	Carbidopa	Replicase polyprotein 1ab	P0DTD1
CHEMBL2354773	Aurothioglucose	Replicase polyprotein 1ab	P0DTD1
CHEMBL55400	Proflavine	Replicase polyprotein 1ab	P0DTD1
CHEMBL1096979	Benserazide	Replicase polyprotein 1ab	P0DTD1
CHEMBL929	Delavirdine	Replicase polyprotein 1ab	P0DTD1
CHEMBL1382627	Sulfadiazine	Replicase polyprotein 1ab	P0DTD1
CHEMBL1401	Nitazoxanide	Spike	P0DTC2
CHEMBL422	Trifluoperazine	Spike	P0DTC2
CHEMBL196	Ascorbic acid	Spike	P0DTC2
CHEMBL1200633	Ivermectin	Spike	P0DTC2
CHEMBL313006	Dalcetrabip	Spike	P0DTC2

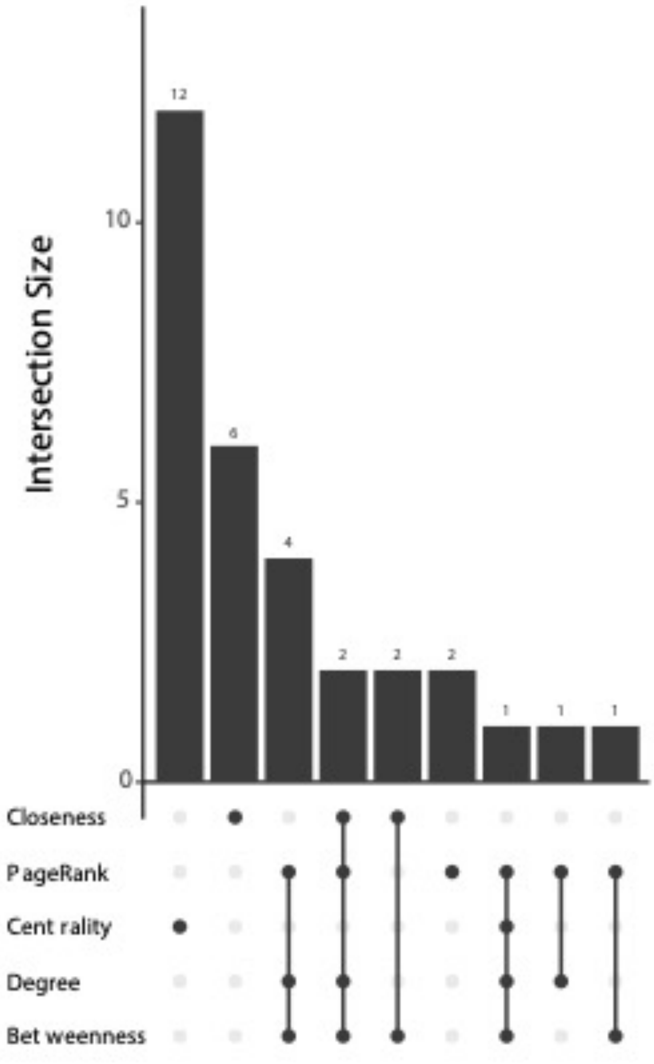

Supplementary Figure S1. Intersection diagram for top 31 proteins according to the topology parameters (closeness, PageRank, centrality, degree, and betweenness) measured for the PPI network. The intersection size describes the number of proteins which meet and/or share top values for each parameter, as indicated by a black circle. For example. There is only one protein in the PageRank and betweenness groups, another different protein in the PageRank and degree groups, and another protein in the PageRank, centrality, degree, and betweenness groups. For further information about the proteins see Supplementary Table S2.

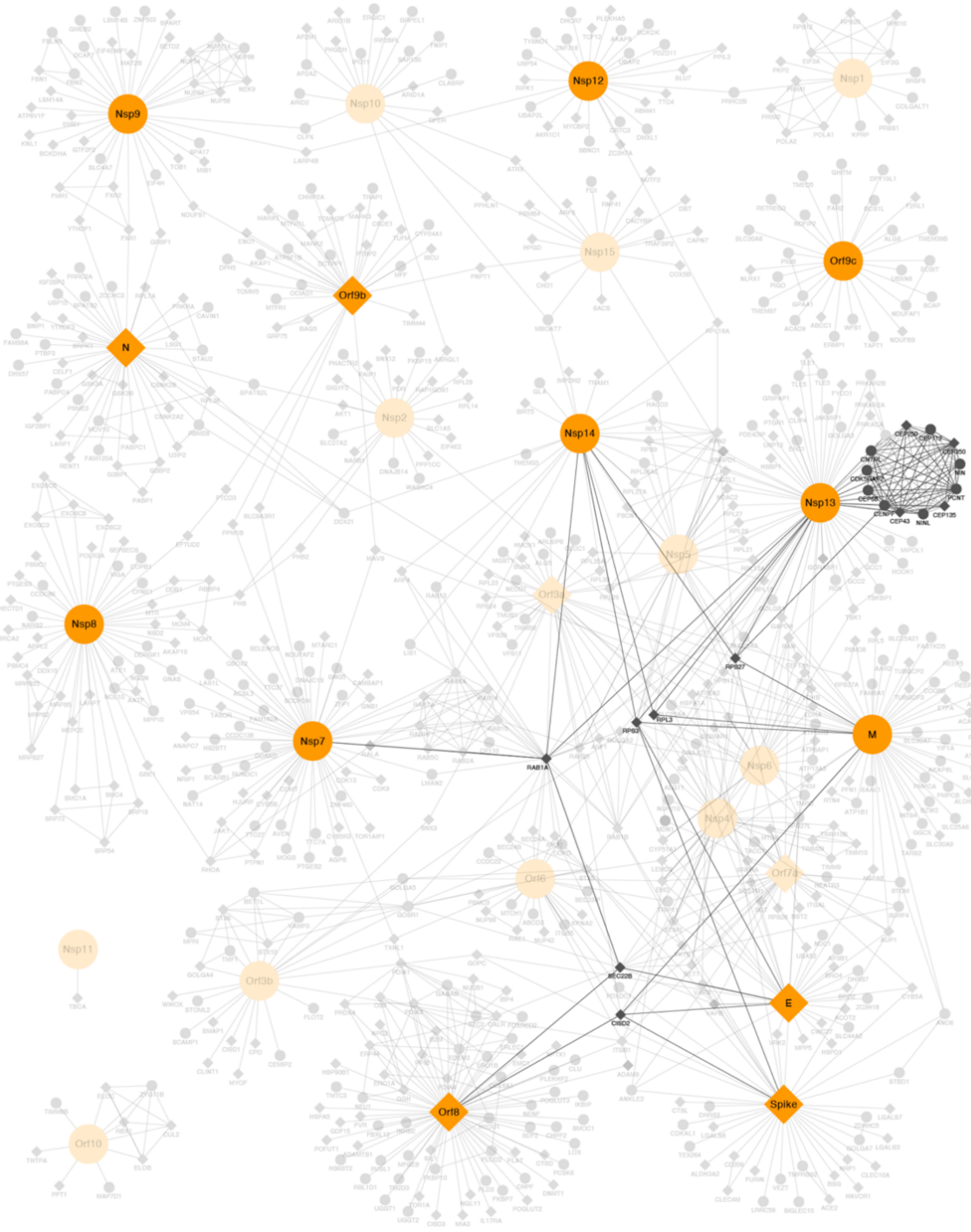

Supplementary Figure S2. Top-proteins identified in the viral-human protein-protein interaction network (PPI). Viral and Human proteins are shown in orange and gray, respectively. Proteins with crystallized structures deposited on the Protein Data Bank are shown as diamonds.

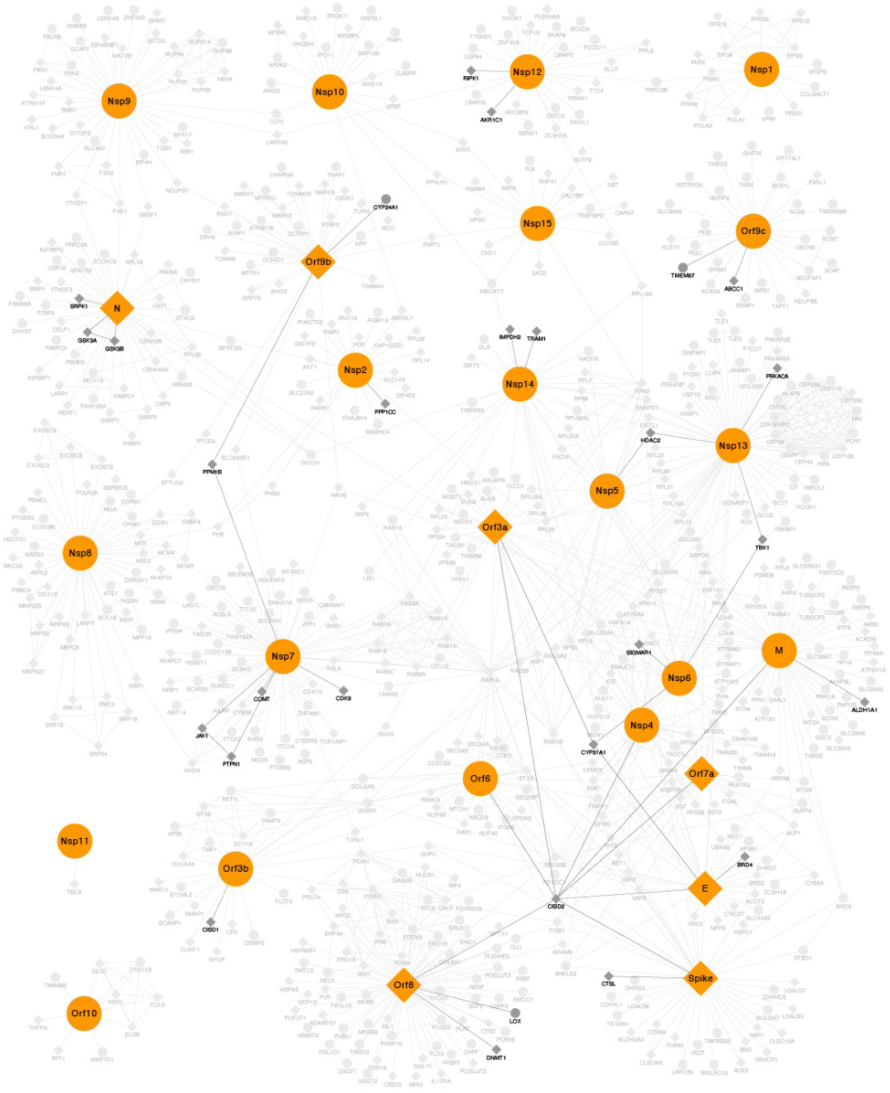

Supplementary Figure S3. Common proteins between the Viral-human Protein-protein (PPI) and the Drug- protein interaction (DPI) networks. Viral and Human proteins are shown in orange and gray, respectively. Proteins with crystallized structures deposited on the Protein Data Bank are shown as diamonds.
